# EnHiC: learning fine-resolution Hi-C contact maps using a generative adversarial framework

**DOI:** 10.1093/bioinformatics/btab272

**Published:** 2021-07-12

**Authors:** Yangyang Hu, Wenxiu Ma

**Affiliations:** Department of Computer Science and Engineering, Riverside, CA 92521, USA; Department of Statistics, University of California Riverside, Riverside, CA 92521, USA

## Abstract

**Motivation:**

The high-throughput chromosome conformation capture (Hi-C) technique has enabled genome-wide mapping of chromatin interactions. However, high-resolution Hi-C data requires costly, deep sequencing; therefore, it has only been achieved for a limited number of cell types. Machine learning models based on neural networks have been developed as a remedy to this problem.

**Results:**

In this work, we propose a novel method, EnHiC, for predicting high-resolution Hi-C matrices from low-resolution input data based on a generative adversarial network (GAN) framework. Inspired by non-negative matrix factorization, our model fully exploits the unique properties of Hi-C matrices and extracts rank-1 features from multi-scale low-resolution matrices to enhance the resolution. Using three human Hi-C datasets, we demonstrated that EnHiC accurately and reliably enhanced the resolution of Hi-C matrices and outperformed other GAN-based models. Moreover, EnHiC-predicted high-resolution matrices facilitated the accurate detection of topologically associated domains and fine-scale chromatin interactions.

**Availability and implementation:**

EnHiC is publicly available at https://github.com/wmalab/EnHiC.

**Supplementary information:**

Supplementary data are available at *Bioinformatics* online.

## 1 Introduction

Recent developments of the high-throughput chromosome conformation capture (Hi-C) techniques (Duan *et al.*, 2010; Lieberman-Aiden *et al.*, 2009; Rao *et al.*, 2014) have enabled us to detect genome-wide chromatin interactions and investigate the organizational principles of the three-dimensional (3D) genome. Studies of Hi-C data have revealed the multi-scale organization of the 3D genome, including active/inactive chromosomal compartments (Lieberman-Aiden *et al.*, 2009), topologically associated domains (TADs) (Dixon *et al.*, 2012) and fine-scale chromatin loops (Rao *et al.*, 2014; Ma *et al.*, 2015). Large-scale chromatin structures, such as compartments and TADs, can be identified from relatively low-resolution (50 kb to 1 Mb) Hi-C contact matrices. However, detecting fine-scale chromatin loops often requires high-resolution (i.e. 10 kb or finer) contact matrices. Moreover, fine-resolution Hi-C data are more compatible with other genomic and epigenomic data, and could therefore facilitate the interrogation of genome regulation and function.

However, high-resolution chromatin contact maps require costly, deep sequencing, and have been achieved in only a limited number of cell lines. For instance, a kilobase-resolution Hi-C map of human lymphoblastoid GM12878 cells required five billion chromatin contacts (Rao *et al.*, 2014). Without sufficient sequencing depth, the observed Hi-C contact maps are often sparse and noisy, which imposes great computational challenges on the identification of chromatin loops between distal regulatory elements and their target genes. Therefore, computational approaches to enhance the resolution of Hi-C contact maps would greatly facilitate the investigation of the 3D genome at a finer scale, and are therefore in great demand.

Several pioneering works on predicting higher-resolution contact frequency matrices from low-resolution Hi-C data have emerged since 2018. The HiCPlus method (Zhang *et al.*, 2018a) was the first attempt to enhance Hi-C data resolution with a convolutional neural network (CNN) by minimizing the L2 mean square error (MSE) loss function. Similar to the image super-resolution approach (Zhang *et al.*, 2018b), HiCPlus extracts hidden features from high-resolution Hi-C matrices in the training process and then predicts high-resolution Hi-C matrices from low-resolution input data. Later, Liu and Wang (2019) proposed the HiCNN model, which employs a more complex (with more than 14 layers) and efficient CNN model with residual learning by utilizing skip connections. However, both HiCPlus and HiCNN use the MSE loss; therefore, they are sensitive to outliers and would result in blurred output when the input Hi-C matrix is sparse.

More recently, several generative adversarial network (GAN) models, such as hicGAN (Liu *et al.*, 2019), Deephic (Hong *et al.*, 2020) and HiCSR (Dimmick *et al.*, 2020), have been proposed to enhance Hi-C matrix resolution. The general GAN framework consists of two neural networks: a generator and a discriminator that contest with each other. In the training step, the generator learns to create a candidate to deceive the discriminator, while the discriminator learns to distinguish the generated candidate from the true data. First, hicGAN (Liu *et al.*, 2019) adopts the SRGAN model (Ledig *et al.*, 2017) in image super-resolution to enhance resolution of Hi-C matrices. The hicGAN model uses a skip-connection network as the generator and replaces the traditional pixel-wise MSE loss with a purely adversarial loss. As a result of minimizing the adversarial loss, hicGAN often misses fine-scale image details. Later, Hong *et al.* (2020) proposed Deephic, a model similar to hicGAN. To recover fine-scale image details, Deephic uses a mixture loss function that consists of the MSE loss, total variation loss, perceptual loss and adversarial loss. The perceptual loss component was derived from the VGG-type model (Simonyan and Zisserman, 2014). However, this perceptual loss causes unwanted natural image textures in the output. Lastly, the HiCSR model (Dimmick *et al.*, 2020) uses a skip-connection network as the generator and a CNN as the discriminator. Their loss function consists of the L1 mean absolute error (MAE) loss, feature loss and adversarial loss. The feature loss was derived from a pre-trained model, which is a denoising autoencoder modified from an image restoration architecture (Mao *et al.*, 2016).

The previously proposed models, hicGAN, Deephic and HiCSR, have demonstrated the power of the GAN framework in predicting high-resolution Hi-C matrices. However, these models treat the Hi-C matrix as a one-channel image and their GAN networks are primarily built on image super-resolution models. As a result, their predictions often contain image artifacts and, therefore, do not accurately represent the underlying chromatin interaction features of the Hi-C data.

To tackle this problem, we developed a new GAN-based model, EnHiC, to enhance the resolution of Hi-C contact frequency matrices. Specifically, we propose a novel convolutional layer (the *Decomposition & Reconstruction Block*, see Methods) that accounts for the non-negative and symmetric properties of Hi-C matrices. In our GAN framework, the generator extracts rank-1 matrix features from multiple scales of low-resolution matrices and predicts the high-resolution matrix via a series of sub-pixel CNN layers (Shi *et al.*, 2016). Accordingly, the discriminator decomposes a high-resolution Hi-C matrix into multiple lower-resolution matrices and extracts the corresponding rank-1 matrix features to determine whether the high-resolution matrix is derived from the generator or the true data.

We evaluated the performance of our EnHiC model using published Hi-C datasets in three human cell lines: GM12878 (lymphoblastoid cells), IMR90 (lung fibroblast cells) and K562 (leukemia cells) (Rao *et al.*, 2014). We demonstrated that EnHiC accurately enhanced the resolution of Hi-C data and achieved high similarity scores with respect to the true high-resolution data, outperforming previously proposed GAN-based models. Using the model trained in one cell type, EnHiC effectively enhanced the resolution of insufficient sequenced Hi-C data in other cell types. In addition, using the EnHiC-enhanced data, we successfully recovered Hi-C-specific features, such as TADs and significant chromatin interactions.

## 2 Materials and methods

### 2.1 Hi-C contact frequency matrix

First, we introduce a few notations regarding the Hi-C contact frequency matrix. A bulk Hi-C experiment characterizes an ensemble of chromatin contacts from thousands or millions of cell nuclei. The raw data generated from the Hi-C experiment can be presented as a non-negative symmetric matrix $C_{N\times N}$, namely the contact frequency matrix, where *N* is the number of fixed-size non-overlapping bins in the genome. Each matrix element *C_ij_* is the observed contact frequency between the genomic loci pair *i* and *j*. A higher contact frequency indicates a smaller spatial distance between a pair of genomic loci in cell nuclei. In short, we refer to the bulk Hi-C contact frequency matrix as the Hi-C matrix.

In our method, we aim to predict high-resolution Hi-C matrices from low-resolution input data. Here, high resolution indicates more chromatin interaction details (i.e. more valid pairs of sequencing reads), rather than a higher dimension of the Hi-C matrix. Given a Hi-C input dataset, it can processed into a matrix of any arbitrary bin size. Therefore, a high-dimensional Hi-C matrix is not always of high resolution. In this work, we refer to the dimension of the Hi-C matrix as its scale. A lower-scale Hi-C matrix has a smaller number of rows and columns.

### 2.2 Overview of the model

In this section, we describe the framework of the EnHiC model. More details of the model are provided in Supplementary Information. EnHiC is based on a GAN framework that contains a generator and a discriminator. Through competition between them, the generator learns to predict high-resolution Hi-C matrices from low-resolution input matrices, while the discriminator distinguishes the generator-predicted high-resolution matrices from real data.

**Fig. 1.**
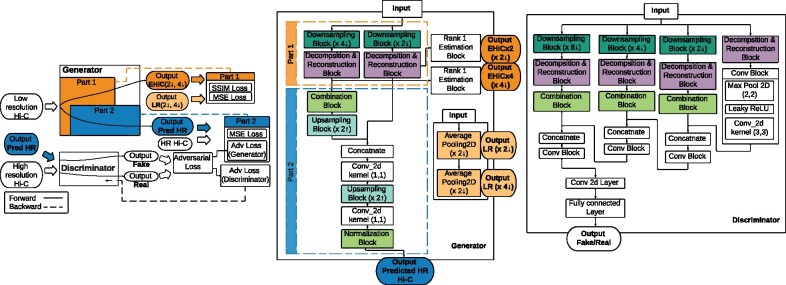
The framework of the EnHiC model. The details of the *Downsampling Block*, *Upsampling Block*, *Combination Block*, *Normalization Block*, *Rank-1 Estimation Block* and *Decomposition & Reconstruction Block* are illustrated in Supplementary Figure S1

The main difference between our model and other GAN-based approaches is that EnHiC exploits the unique properties of the Hi-C matrix and treats it as a multi-scale interaction contact map instead of a pure image. Specifically, EnHiC extracts rank-1 matrix features from low-resolution input data at multiple scales and learns to enhance the matrix resolution using these estimated rank-1 features. The overview of the EnHiC framework is illustrated in Figure 1.

#### 2.2.1 Decomposition & reconstruction block

A key component in our model is the *Decomposition & Reconstruction Block*, as illustrated in Figure 1 and Supplementary Figure S1.

In our model, we represent a Hi-C matrix as a multi-channel image (i.e. a tensor). Let $c_{\text{in}}$ and $c_{\text{out}}$ be the number of input and output channels, respectively. The input and output tensors are denoted by $X\in R^{N\times N\times c_{\text{in}}}$ and $\hat{X}\in R^{N\times N\times c_{\text{out}}}$, where *N* is the dimension of the Hi-C matrix. The *Decomposition & Reconstruction Block* contains three layers:

- The decomposition layer, which passes **X** into a convolutional layer with kernel (1,N). In contrast to the traditional convolutional layer, the kernel is a vector rather than a square matrix. The length of the kernel vector is the same as the height/width of the input tensor. Hence, the kernel only moves in one direction, and the number of shared parameters for this convolutional layer is $N\times c_{\text{in}}\times c_{\text{out}}$. The resulting tensor is denoted by $U=[u_{1},\ldots ,u_{c_{\text{out}}}]\in R^{N\times 1\times c_{\text{out}}}$, which represents the rank-1 features of the input data.
- The weighting layer, which scales the feature tensor as $V=Uww^{⊤}$, where the weight vector $w={\left[w_{1},\ldots ,w_{c_{\text{out}}}\right]}^{T}$ is a learnable parameter.
- The reconstruction layer, which constructs the output tensor $\hat{X}$ using the weighted rank-1 features. For each channel *k*, we have a rank-1 matrix ${\hat{X}}_{::k}=v_{k}v_{k}^{⊤}$, where $k=1,\ldots ,c_{\text{out}}$.

#### 2.2.2 Generator

The generator consists of two parts: (i) extracting rank-1 matrix features from low-resolution input matrices at multiple scales and (ii) enhancing Hi-C matrix resolution using the multi-scale features learned in the first part. The overview of the generator framework (*G*_1_ in the orange dashed box and *G*_2_ in the blue dashed box) is shown in Figure 1.

Because the low-resolution input matrix is often sparse, we first downscale the matrix to enhance its signal. The downscaling operation is achieved by shrinking the size of the matrix by an average-pooling layer. In our experiments, we aim to enhance the resolution of the Hi-C matrix by a factor of 16, which is equivalent to scaling up the matrix by a factor of 4 (i.e. multiplying both the height and width of the matrix by 4). Therefore, in our model, we generate two downscaled matrices by factors of 2 and 4 [denoted as LR(×2↓) and LR(×4↓), respectively]. We use LR(×2↓) and LR(×4↓) as the ground truth to assist in the estimation of the rank-1 matrix features at the corresponding scales. Note that in our EnHiC framework, the number of downscaling operations can be adjusted for different applications. For instance, if we aim to enhance the Hi-C resolution by a factor of 100, it is recommended to include additional levels of downscaled matrices (and accordingly, more *Decomposition & Reconstruction Blocks*) to facilitate a better estimation of matrix features.

The first part of the generator (*G*_1_) extracts multi-scale rank-1 features from the low-resolution input matrix. First, it transforms the input matrix (*N *×* N*) into a tensor ($\frac{N}{r}\times \frac{N}{r}\times r^{2}$) using a space-to-depth layer (TensorFlow built-in function). The space-to-depth layer permutes the spatial blocks of the input matrix into the depth dimension without any loss of information. Then, a multi-channel image (tensor) is subsequently processed through the *Decomposition & Reconstruction Block* and its rank-1 features are extracted. Note that the input Hi-C matrix is symmetric and non-negative, and our rank-1 approximations retain the symmetric and non-negative properties of the data. In our framework, we extract the rank-1 features for two different scales (r=2 and 4, respectively), and the two estimation matrices, denoted as EHiC(×2↓) and EHiC(×4↓), are compared against the true data, as shown in Figure 1.

The second part of the generator (*G*_2_) recombines the rank-1 features from multiple scales and enhances the matrix resolution through a series of *Upsampling Blocks*. The *Upsampling Block* contains a sub-pixel convolutional layer (Shi *et al.*, 2016) that upscales the previously learned features in low-resolution space to a high-resolution output. The upscaled tensor is subsequently averaged with its transpose to reinforce the symmetric property of the output matrix. In concert with the two *Decomposition & Reconstruction Blocks* in the first part, we have two *Upsampling Blocks*, each of which upscales the matrix dimension by a factor of 2 (i.e. enhancing the data resolution by a factor of 4). Therefore, the final output matrix has an enhanced resolution by a factor of 16 compared to the low-resolution input matrix. Details of the *Upsampling Block* are illustrated in Supplementary Figure S1.

#### 2.2.3 Loss functions of the generator

The objective of the generator is to estimate the rank-1 features at multiple scales and to enhance resolution of the input matrix. Therefore, we design two loss functions for these two tasks separately. Although the extraction of rank-1 features can be obtained using a pre-trained model, we combine it in the generator network so that we can reuse the intermediate rank-1 feature data in the training process. Therefore, the generator has two loss functions and two back-propagation steps to update their associated parameters separately.

***Loss function for low-resolution approximation (rank-1 matrix features)*** Inspired by NMF, the approximate low-resolution Hi-C matrix is calculated as a combination of rank-1 matrices. To estimate these rank-1 matrices, we include both pixel-wise MSE loss and structural dissimilarity (DSSIM) measures in the loss function. The DSSIM metric is derived from the structural similarity (SSIM) metric (Wang *et al.*, 2004) to quantify the perceptual differences between two images. Specifically, $\text{DSSIM}=\frac{1-\text{SSIM}}{2}\in [0,1]$. As described above, the generator may involve more than one downscaled representation of the low-resolution input, so we denote the factor set as $f=[f_{1},\ldots ,f_{K}]$ and the corresponding weights for the downscaled matrices as $w=[w_{1},\ldots ,w_{K}]$, where $w_{k}=\frac{f_{k}^{2}}{\sum_{k}f_{k}^{2}}$. The loss function of rank-1 feature extraction is:

$$\ell_{G_{1}}(\hat{I},I)=\sum_{k=1}^{K}w_{k}\left[\ell_{\text{MSE}}(\hat{I},I)+\text{DSSIM}(\hat{I},I))\right].$$

In our application, we downscale the low-resolution input matrix by two different factors. Hence, *K *=* *2, $f_{1}=2$ and $f_{2}=4$.

***Loss function for high-resolution enhancement*** In the second part of the generator, we feed the rank-1 matrix features extracted from multiple downscaled low-resolution data into several sub-pixel layers to enhance matrix resolution. The loss function for the prediction of a high-resolution matrix consists of the pixel-wise MSE loss and the adversarial loss:

$$\ell_{G_{2}}(I_{\text{SR}},I_{\text{HR}})=\alpha_{0}\ell_{\text{MSE}}(I_{\text{SR}},I_{\text{HR}})+\alpha_{1}\ell_{\text{adv}},$$

where the *α*_0_ and *α*_1_ are hyperparameters.

The adversarial loss $\ell_{\text{adv}}$ is a crucial part of the GAN framework that connects the generator and discriminator networks. For the generator, minimizing the loss is equivalent to minimizing the binary cross-entropy loss between the true label (**y**) and the prediction (**x**) of generated Hi-C matrices by the discriminator. That is, $\ell_{\text{bce}}(y,x)=-\frac{1}{N}\sum_{n=1}^{N}\left(y_{n}\cdot \;\text{log}(x_{n})+(1-y_{n})\cdot \;\text{log}(1-x_{n})\right)$. To disorient the discriminator, all labels of the predicted matrices are set to true. More details on the adversarial loss are discussed in Section 2.2.5.

$$\begin{array}{c} \ell_{\text{adv}}=\ell_{\text{bce}}(1,D(G(I_{\text{LR}})))\\ =-\text{log}(D(I_{\text{SR}})) \end{array}$$

#### 2.2.4 Discriminator

The discriminator aims to differentiate between high-resolution predictions from the generator and real high-resolution data. In our EnHiC model, the discriminator shares the same strategy of the multi-scale rank-1 approximation as the generator, as illustrated in Figure 1. First, the input matrix is converted to multiple downscaled tensors by space-to-depth layers (in the *Downsampling Block*) and the rank-1 matrix features are subsequently extracted from each of the downscaled tensors (in the *Decomposition & Reconstruction Block*). In our design, we extract rank-1 features from the original matrix as well as three downscaled matrices (by a factor of 2, 4 and 8, respectively). Second, these rank-1 matrix features are passed into a cascade of *Convolutional Blocks* to detect latent features at multiple resolutions. As shown in Figure 1, each *Convolutional Block* includes a Leaky ReLU layer, a max-pooling layer and a 2D convolution layer. After pooling and convolution, the dimensions of rank-1 matrix features are reduced by a factor of 2. These higher-resolution features are then concatenated with lower-resolution features and passed into the subsequent *Convolutional Block*. Finally, after a fully connected layer, the discriminator outputs the probability that the input is real, that is, the true high-resolution data rather than a prediction from the generator.

#### 2.2.5 Loss function of the discriminator

In the training process, the generator and discriminator compete with each other and are connected by a MinMax loss. The generator tries to minimize the following function while the discriminator attempts to maximize it:

$${\min_{G}}_{}{\max_{D}}_{}E_{I_{\text{HR}}}\left[\text{log}\;(D(I_{\text{HR}}))\right]+E_{I_{\text{LR}}}\left[\text{log}\;(1-D(G(I_{\text{LR}})))\right],$$

where D(·) is the estimated probability by the discriminator. $E_{I_{\text{HR}}}$ is the expected value over all true instances. $G(I_{\text{LR}})$ is the generator’s output when fed with the low-resolution Hi-C matrix $I_{\text{LR}}$, which is also called the super-resolution Hi-C matrix $I_{\text{SR}}$. $E_{I_{\text{LR}}}$ is the expected value over all generated instances.

The GAN framework has two adversarial loss functions: one for generator training (as discussed in Section 2.2.3) and one for discriminator training. The discriminator aims to maximize $E_{I_{\text{HR}}}\left[\text{log}\;(D(I_{\text{HR}}))\right]+E_{I_{\text{LR}}}\left[\text{log}\;(1-D(G(I_{\text{LR}})))\right]$. Thus, the adversarial loss of the discriminator can be expressed as a combination of two binary cross-entropy losses:

$$\begin{array}{c} \ell_{D}=\ell_{\text{bce}}(1,D(I_{\text{HR}}))+\ell_{\text{bce}}(0,D(G(I_{\text{LR}})))\\ =-\text{log}(D(I_{\text{HR}}))-\text{log}(1-D(I_{\text{SR}})) \end{array}$$

## 3 Results

### 3.1 EnHic accurately predicts high-resolution Hi-C matrices

First, we sought to evaluate the enhancement capability of our EnHiC model against two other GAN-based models, Deephic and HiCSR. It has been shown that Deephic and HiCSR outperformed previously proposed models, including HiCPlus, HiCNN and hicGAN. Therefore, these models were not included in our evaluation. All three models, EnHiC, Deephic and HiCSR, were trained to predict a high-resolution (10 kb) Hi-C matrix from a low-resolution (40 kb) Hi-C matrix. In other words, the desired resolution enhancement factor was 16.

#### 3.1.1 Data preprocessing

In our validation experiments, we used three published Hi-C datasets in different human cell lines: GM12878 (lymphoblastoid cells), IMR90 (lung fibroblast cells) and K562 (leukemia cells) (Rao *et al.*, 2014). Among them, the GM12878 dataset has the highest number of chromatin contacts (2.88 billion), followed by IMR90 (0.76 billion) and K562 (0.62 billion) (Supplementary Table S1). High-resolution (10 kb) Hi-C matrices were obtained from the cooler database (Abdennur and Mirny, 2020). Low-resolution Hi-C matrices were generated using a random downsampling procedure. Here we used the default downsampling ratio of 16. In other words, the sequencing depth in the resulting low-resolution matrices was 1/16 of the high-resolution data.

First, we trained the three models (EnHiC, Deephic and HiCSR) on the most deeply sequenced Hi-C data generated from GM12878 cells. We used chromosomes 1-16 for training, chromosomes 17 and 18 for hyperparameter tuning, and chromosomes 19–22 and X for evaluation. After model training in the GM12878 data, we applied the three methods to the IMR90 and K562 data to investigate the enhancement performance across different cell types.

The raw Hi-C matrix contains various types of technical and biological biases. Therefore, normalization is an essential step in Hi-C data analysis. Many normalization methods based on matrix-balancing approaches have been proposed (Imakaev *et al.*, 2012; Knight and Ruiz, 2013; Kumar *et al.*, 2017; Servant *et al.*, 2015). In the EnHiC model, we employ the Sequential Component Normalization (SCN) method (Servant *et al.*, 2015) to normalize the input Hi-C matrix. The Deephic and HiCSR models do not require Hi-C-specific normalization of the input matrix. Instead, Deephic uses the min-max normalization to scale the input data. HiCSR first conducts a log1p transformation (i.e. log(1+x)) and then a min-max normalization of the input data.

After normalization, the intra-chromosomal Hi-C matrices were divided into small pieces (submatrices of size *n *×* n*) for both training and testing. Here, we set *n *=* *400. Specifically, EnHiC first divides the Hi-C matrix into non-overlapping submatrices of size $\frac{n}{2}\times \frac{n}{2}$ and then combines two diagonal submatrices with their off-diagonal interacting submatrix to form an *n *×* n* matrix. This operation ensures that the resulting submatrices are symmetric. Deephic divides the Hi-C matrix into non-overlapping submatrices of size 40 × 40. HiCSR divides the Hi-C matrix into partially overlapping submatrices of size 40 × 40 with a step size of 28 × 28. Therefore, the input submatrices are of size 40 × 40 and the output submatrices are of size 28 × 28. Because the average TAD size is less than 1 Mb and most of the significant interactions are located inside TADs, we omitted submatrices with the genomic distances greater than 2 Mb.

#### 3.1.2 Training and prediction

The EnHiC model was implemented in Python 3 with TensorFlow2; and the source code is available at https://github.com/wmalab/EnHiC. Both the training and prediction processes of the three assessed models were conducted on Intel Haswell CPU and NVIDIA Tesla K80 GPU with 128 GB of memory. For EnHiC, the number of epochs for training was set to 300 with parameters $\alpha_{0}=10$ and $\alpha_{1}=0.1$. The runtime of the training process was approximately 85 hours (17 min per epoch). More training details, including the configuration and visualization generated by TensorBoard, are available in Supplementary Information. The runtimes for HiCSR (500 epochs) and Deephic (800 epochs) were approximately 2 to 4 days.

#### 3.1.3 Model validation and evaluations in GM12878 data

After the training step, we first applied the three models (EnHiC, Deephic and HiCSR) to the evaluation set (chromosomes 19-22 and X) in human GM12878 data to enhance the resolution of low-resolution Hi-C matrices (downsampled from high-resolution Hi-C matrices by a factor of 16). We denote the 10 kb high-resolution Hi-C matrices obtained from the cooler database as the ground truth.

For each chromosome, we assembled the predicted submatrices into one intra-chromosomal matrix. Because different models use different normalization procedures, it is necessary to reverse the normalizations to facilitate a fair comparison with the same ground truth. Denote the model output as **X**, and de-normalized result as $\tilde{X}$.

- Deephic uses the min-max normalization. Hence, the reversion is $\tilde{X}=\max X+\min$, where max and min are maximal and minimal values in the ground truth, respectively.
- HiCSR uses both the log1p transformation and the min-max normalization. Therefore, the reversion is $\tilde{X}=e^{\left(\max X+\min\right)}-1$, where max and min are the maximal and minimal log1p values in the ground truth.
- EnHiC uses the SCN normalization, therefore the reversion is $\tilde{X}=X⊘bb^{⊤}$, where **b** is the bias vector estimated from the ground truth using the SCN method and ⊘ is the element-wise division. In the form of each element, we have ${\tilde{X}}_{ij}=\frac{X_{ij}}{b_{i}b_{j}}$.

After reverse normalization, we evaluated the prediction results of the three models with the ground truth using four metrics: two classic pixel-wise numeric errors (MAE and MSE) and two Hi-C-specific similarity metrics: HiCRep (Yang *et al.*, 2017) and GenomeDISCO (Ursu *et al.*, 2018). Supplementary Table S2 summarizes the MAE and MSE measurements of the EnHiC, Deephic and HiCSR predictions. Overall, EnHiC achieved the best performance with the lowest MAE and MSE errors. We noticed that MAE and MSE errors were inflated in Deephic and HiCSR predictions. This is likely due to the reverse normalization procedure, where the MAE and MSE errors were amplified by the *max* value and exponential operation. Therefore, the MAE and MSE metrics were not effective in assessing the performance of the Hi-C enhancement. We present the results for reference because MAE is a component of the loss function in the HiCSR model, and MSE is included in the loss functions in both EnHiC and Deephic.

In addition to the MAE and MSE metrics, we also considered two popular similarity measurements specifically designed for assessing reproducibility of Hi-C matrices, HiCRep (Yang *et al.*, 2017) and GenomeDISCO (Ursu *et al.*, 2018). HiCRep calculates a stratum-adjusted correlation coefficient (SCC) between two Hi-C matrices. The resulting SCC values range from –1 to 1, where a larger SCC value indicates a higher similarity between the two matrices. GenomeDISCO treats the Hi-C matrix as a network; it applies random walks on the network to smooth the data and then calculates a reproducibility score at multiple scales. Similar to HiCRep, GenomeDISCO scores also range from –1 to 1, where higher scores representing the higher reproducibility. Besides HiCRep and GenomeDISCO, HiC-Spector (Yan *et al.*, 2017) is another Hi-C reproducibility metric. HiC-Spector applies the adjacency matrix to impute missing values and then calculates a similarity score between two full matrices. In our experiments, since we only predicted a strip of data in the full matrix (i.e. submatrices with genomic distances shorter than 2 Mb), HiC-Spector is not applicable in our evaluation.

Table 1 summarizes the HiCRep and GenomeDISCO evaluation results of EnHiC, Deephic and HiCSR. As shown in Table 1, The HiCRep SCC scores were greater than 0.94 for all three methods, indicating that their high-resolution predictions are very similar to the ground truth. Among them, our EnHiC model achieved the highest HiCRep SCC values and GenomeDISCO scores for all five test chromosomes. These results demonstrated that EnHiC can accurately and robustly enhance the resolution of Hi-C matrices and outperformed existing GAN-based models.

**Table 1.** Evaluation of high-resolution Hi-C matrices predicted by EnHiC, Deephic and HiCSR

	HiCRep			GenomeDISCO		
Chromosome	EnHiC	Deephic	HiCSR	EnHiC	Deephic	HiCSR
19	**0.972**	0.942	0.970	**0.83**	0.768	0.677
20	**0.972**	0.941	0.967	**0.837**	0.777	0.65
21	**0.973**	0.966	0.968	**0.816**	0.771	0.636
22	**0.978**	0.974	0.973	**0.844**	0.786	0.716
X	**0.949**	0.930	0.945	**0.781**	0.743	0.639

*Note*: Three models are evaluated on chrosomomes 19-22 and X in human GM12878 Hi-C data. Each model prediction result is compared against the ground truth, and the HiCRep and GenomeDISCO scores are calculated. The highest HiCRep and GenomeDISCO scores are highlighted in bold.

#### 3.1.4 Performance on IMR90 and K562 data

In the previous section, we have demonstrated the capability of EnHiC in recovering high-resolution Hi-C matrices from low-resolution input data. We then asked whether EnHiC can enhance Hi-C matrix resolution across different cell types. Toward this goal, we applied three models (EnHiC, Deephic and HiCSR) that were previously trained on the deeply sequenced GM12878 (lymphoblastoid cells) dataset to two other less-sequenced Hi-C datasets: IMR90 (lung fibroblast cells), and K562 (leukemia cells). The same data preprocessing was performed in each cell type; and HiCRep and GenomeDISCO similarity scores were calculated to evaluate the model predictions.

**Fig. 2.**
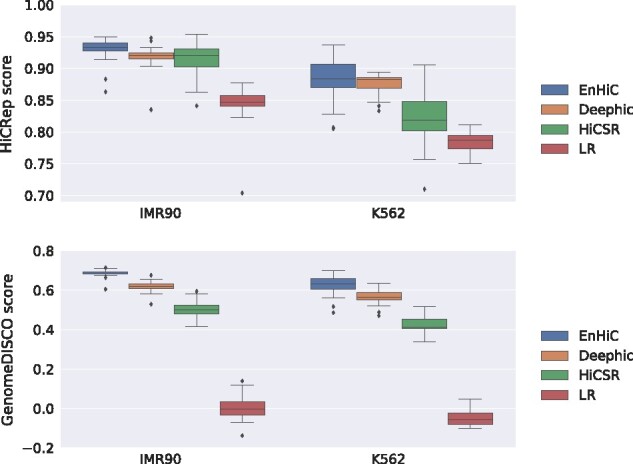
Evaluation of high-resolution Hi-C matrix predictions by EnHiC, Deephic and HiCSR on human IMR90 and K562 Hi-C data (23 chromosomes). The models are first trained on GM12878 data and then applied to the other cell types. Each prediction result is compared against the ground truth, and the HiCRep and GenomeDISCO similarity scores are reported. Each box represents similarity scores of 23 chromosomes (1–22 and X). Low-resolution (LR) input data are included as the baseline

Figure 2 illustrates the cross-cell-type performance of EnHiC, Deephic and HiCSR. Overall, EnHiC outperformed both Deephic and HiCSR with the highest HiCRep and GenomeDISCO scores in both IMR90 and K562 datasets. We observed that the HiCRep and GenomeDISCO similarity scores were relatively lower than the ones previously obtained from GM12878 data, but they were significantly higher that the baseline (low-resolution input data). In addition, the performance of all three models were slightly better in IMR90 than K562. This is likely due to the relatively higher sequencing depth in the IMR90 data (Supplementary Table S1). Taken together, these results indicated that EnHiC can effectively recover high-resolution matrices from insufficiently sequenced Hi-C data across cell types.

#### 3.1.5 Performance on different downsampling ratios

In the training process, we generated low-resolution Hi-C matrices that were 16× downsampled from high-resolution ground truth, i.e. the sequencing depth of the low-resolution input data was 1/16 of the high-resolution data. We set the downsampling ratio at 16 to facilitate a fair comparison with previously published methods (Deephic and HiCSR). Although being trained by 16× downsampled data, our EnHiC model is flexible and can be applied to low-resolution data with much less sequencing depth. Next, we sought to investigate the performance of our model using low-resolution input data generated with different downsampling ratios.

In this experiment, we generated low-resolution input data at six different downsampling ratios (4, 8, 16, 32, 48 and 64). We trained three models (EnHiC, Deephic, HiCSR) on the human GM12878 data using the same training set (chromosomes 1–16) and validation set (chromosomes 17–18) at 16× downsampled ratio as previously described. We then evaluated the model performance using all 23 chromosomes at six different downsampled ratios, except for the 16× downsampled data where the 18 training and validation chromosomes were excluded.

**Fig. 3.**
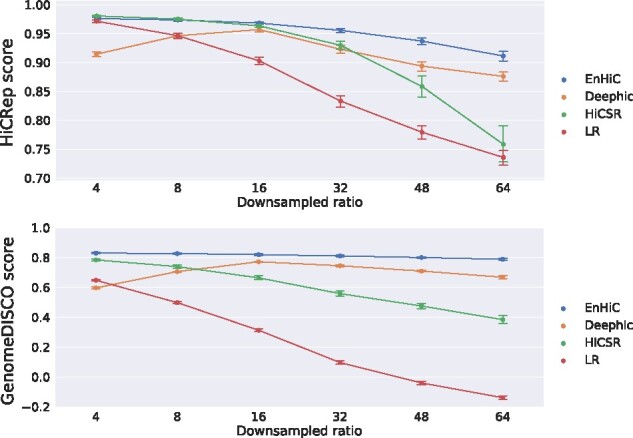
Performance of high-resolution Hi-C matrix predictions by EnHiC, Deephic and HiCSR on GM12878 data at various downsampling ratios (4, 8, 16, 32, 48 and 64). Each prediction result is compared against the ground truth; and the HiCRep and GenomeDISCO reproducibility scores are reported. The mean values and error bars are calculated using scores from 23 chromosomes (1-22 and X). Low-resolution (LR) input data are included as the baseline

As shown in Figure 3, the HiCRep and GenomeDISCO similarity scores of low-resolution input baseline decreased sharply as the downsampling ratio increased. Notably, our EnHiC model robustly and stably recovered high-resolution Hi-C matrices from low-resolution input data with large downsampled ratios. Moreover, EnHiC achieved higher HiCRep and GenomeDISCO scores than DeepHiC and HiCSR at almost all downsampled ratios. Although HiCSR performed slightly better than EnHiC by the HiCRep metric when the downsampling ratio was 4, its performance dropped sharply when the downsampling ratio increased. This is probably due to the pre-trained denoise model used in the loss function of HiCSR. Collectively, these results demonstrated that EnHiC can successfully predict high-resolution Hi-C matrices from insufficiently sequenced low-resolution data.

### 3.2 EnHiC facilitates accurate detection of TADs

TADs are functional units of chromatin, where chromatin interactions are observed more frequently within TADs than outside TADs. TAD boundaries are largely conserved across cell types and are enriched with CTCF and other chromatin-binding proteins (Dixon *et al.*, 2012). To investigate whether high-resolution enhancing methods promote TAD detection, we compared the TADs identified from high-resolution predictions by EnHiC, Deephic and HiCSR, with the TADs identified from the true high-resolution data.

Several computational methods exist for detecting TADs in Hi-C contact maps. Here, we used the hicFindTADs method in the HiCExplorer package (Wolff *et al.*, 2018). We calculated Jaccard scores to assess the consistency between TADs detected from model predictions and TADs detected from true high-resolution (HR) data. The Jaccard score measures the similarity between two sets and is defined as the ratio of the intersection size over the union size. Jaccard score has been commonly used to quantify similarities of TAD and chromatin loop detections (Forcato *et al.*, 2017; Hong *et al.*, 2020). Here we calculated Jaccard scores of TAD boundaries and allowed the boundaries to be shifted within 5 bins between the two sets.

$$\text{Jaccard}\;\text{score}=\frac{{\text{TAD}}_{\text{HR}}\cap {\text{TAD}}_{\text{prediction}}}{{\text{TAD}}_{\text{HR}}\cup {\text{TAD}}_{\text{prediction}}}$$

**Fig. 4.**
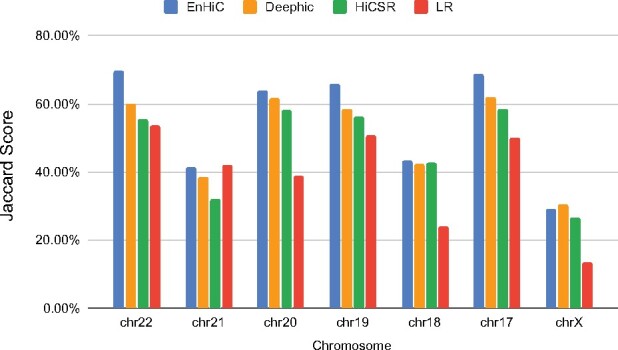
The Jaccard scores of TADs. TADs detected from high-resolution predictions by EnHiC, Deephic and HiCSR were compared with TADs detected from real high-resolution (10 kb) Hi-C data, for chromosomes 17–22 and X. TAD detection results from low-resolution (LR) input data were also included

**Fig. 5.**
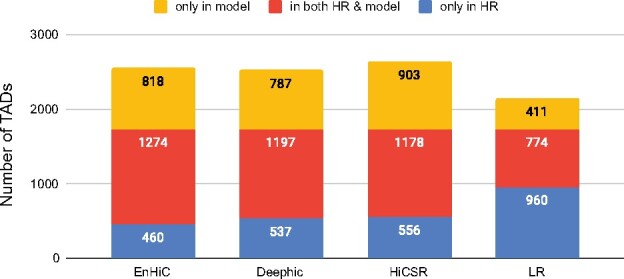
Numbers of TADs detected by each model. The results of seven chromosomes (17–22 and X) are summed. The red bars represent common TADs in both the true high-resolution (HR) matrices and model predictions. The blue (yellow) bars represent unique TADs detected only in the HR (predicted) matrices

Figure 4 illustrates the Jaccard score evaluation of various methods in the validation dataset (chromosomes 17 and 18) and the test dataset (chromosomes 19–22 and X). The TADs detected from low-resolution input matrices were also included as baselines. Overall, EnHiC promoted accurate TAD detection; and the identified TADs were highly consistent with the ones identified from the true high-resolution data. In most cases, except for chromosome 21, high-resolution predictions from GAN-based models resulted in more accurate TAD detection than low-resolution input matrices (Fig. 5). Overall, EnHiC yielded the highest Jaccard scores for five out of seven chromosomes, and outperformed both Deephic and HiCSR.

We also characterized the ChIP-seq profiles of several chromatin structural proteins and histone marks at the detected TAD boundaries in EnHiC-predicted matrices (Supplementary Fig. S10). Consistent with the previous findings (Dixon *et al.*, 2012), we observed that CTCF, members of the cohesin complex (SMC3 and RAD21), RNA polymerase PolII binding and H3K4me3 and H3K27me3 histone modifications were enriched at TAD boundaries, whereas H3K9me3 was depleted at such boundaries.

**Fig. 6.**
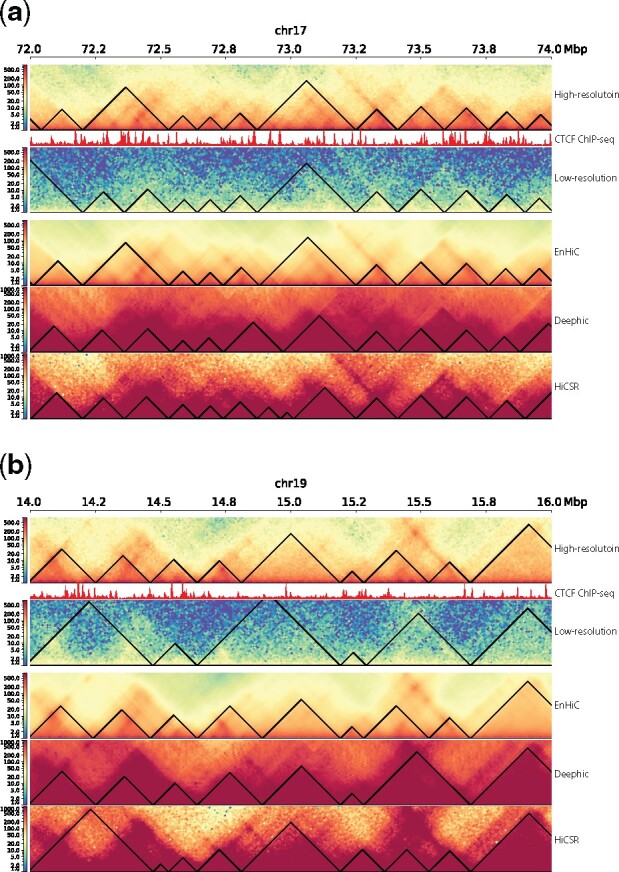
Examples of TAD detection results. (**a**) Chromosome 17 from 72Mbp to 74Mbp, (**b**) Chromosome 19 from 14Mbp to 16Mbp. TADs were identified using HiCExplorer. From top to bottom: true high-resolution (10 kb) Hi-C data, CTCF ChIP-seq signal, low-resolution (40 kb) input Hi-C data and high-resolution predictions from EnHiC, Deephic and HiCSR. For each Hi-C matrix, the heatmap of close-to-diagonal region is displayed with the color key from low (blue) to high (red) interaction frequencies. TADs are identified using HiCExplorer, and marked as black triangles

We further examined TAD detection results in two local regions (chr17:72–74Mbp and chr19:14–16Mbp), as illustrated in Figure 6. The low-resolution input matrices are sparse and noisy; therefore, the detected TADs are often merged or split. Our EnHiC model accurately predicted high-resolution matrices from low-resolution input data. As a result, the TADs detected from EnHiC predictions were in agreement with the TADs from the true high-resolution data in both examples. We observed that both Deephic and HiCSR predictions overinflated the contact frequencies and Deephic predictions contained unwanted image textures, thereby resulting in inaccurate TAD detection.

### 3.3 EnHiC-predicted high-resolution matrices promote precise identifications of significant chromatin interactions

Next, we investigated whether the EnHiC-predicted high-resolution Hi-C data could facilitate the identification of fine-scale chromatin loops. We applied Fit-Hi-C (Ay *et al.*, 2014) to identify significant interactions within 1 Mb genomic distances and compared the overlaps between the real and predicted Hi-C matrices. The Jaccard score was used to assess consistency between the significant interactions in the two matrices.

**Fig. 7.**
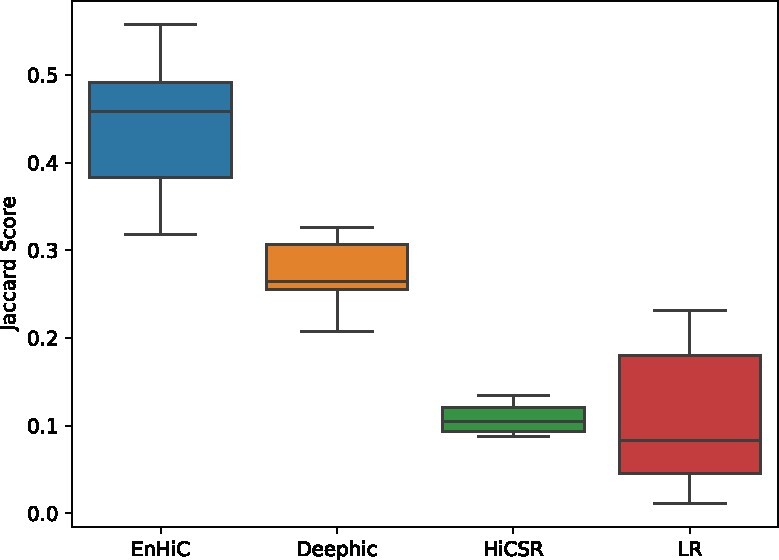
The Jaccard scores of significant interactions between the true high-resolution Hi-C and model predictions. The results from low-resolution (LR) input data were included as baseline. Each box depicts the Jaccard scores of seven chromosomes (17–22 and X)

As shown in Figure 7, EnHiC evidently outperformed the other two GAN-based prediction models with significantly higher Jaccard scores (*t*-tests, *P*-values: $2.57\times 10^{-4}$ (EnHiC versus Deephic), $1.23\times 10^{-7}$ (versus HiCSR) and $4.98\times 10^{-6}$ (versus LR)). The low-resolution Hi-C input matrices lack sufficient sequencing depth; therefore, they are not suitable for the identification of fine-scale chromatin interactions, especially when the genomic distance increases (Supplementary Fig. S11).

**Fig. 8.**
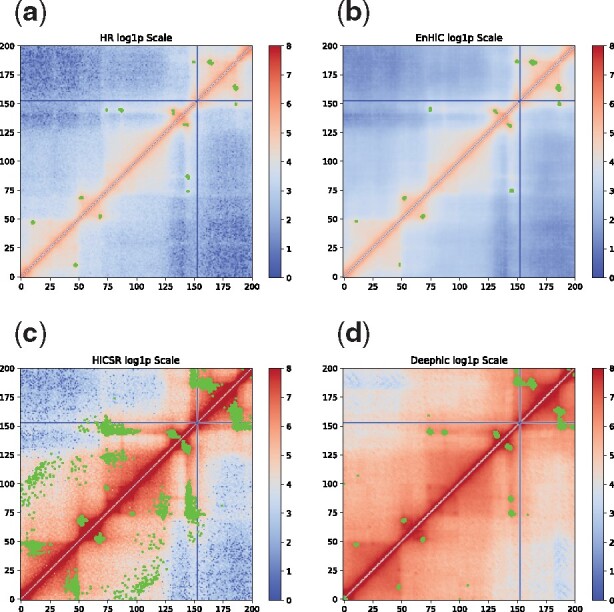
Significant chromatin interactions identified in chromosome 17 from 32Mbp to 34Mbp. (**a**) High resolution (HR) Hi-C at 10 kb, (**b**) EnHiC prediction, (**c**) HiCSR prediction, and (**d**) Deephic prediction. Significant interactions were identified using FitHiC and are highlighted in green. Hi-C matrices are plotted on a log1p scale

**Fig. 9.**
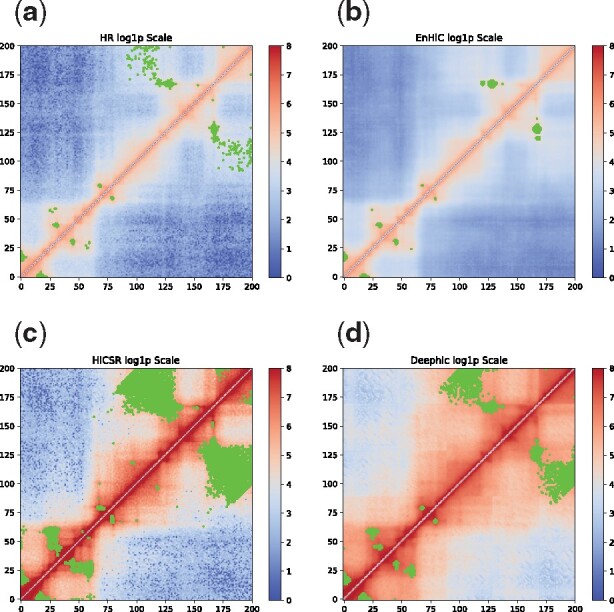
Significant chromatin interactions identified in chromosome 19 from 14Mbp to 16Mbp. (**a**) High resolution (HR) Hi-C at 10 kb, (**b**) EnHiC prediction, (**c**) HiCSR prediction, and (**d**) Deephic prediction. Significant interactions were identified using FitHiC and are highlighted in green. Hi-C matrices are plotted on a log1p scale

We further looked at two example regions, chromosome 17:32–34Mbp (Fig. 8) and chromosome 19:14–16Mbp (Fig. 9). As demonstrated in both regions, EnHiC successfully recovered the high-resolution matrices and produced highly similar chromatin loop identifications as those identified from real high-resolution data. As previously observed, Deephic and HiCSR tended to overinflate the contact matrix, thereby leading to a large number of false discoveries of significant interactions. The high false discovery rate is likely due to the preprocessing procedures or loss functions in these models. For example, HiCSR uses a log1p transformation in its preprocessing step, which may inflate low contact frequencies. In addition, Deephic uses a perceptual loss; as a result, its predictions contained unwanted image textual artifacts. Our EnHiC model is specifically designed to account for the unique data properties in the Hi-C matrix; therefore, the EnHiC-predicted matrices faithfully present high-resolution details in the Hi-C matrix.

## 4 Discussion and conclusions

In this study, we proposed a generative adversarial framework, EnHiC, for predicting high-resolution Hi-C matrices from low-resolution input data. Specifically, high-resolution enhancement is achieved through the extraction of rank-1 matrix features from multi-scale low-resolution input samples and subsequent upsampling processes via sub-pixel CNN layers.

Existing resolution-enhancement models, such as Deephic and HiCSR, treat Hi-C matrices as single-channel images, and leverage on the established neural networks of image super-resolution models. Although such models can produce super-resolution Hi-C matrices, their predictions often overinflate the Hi-C matrix features and sometimes contain unwanted natural image artifacts. Unlike other models, our EnHiC model utilizes the unique properties of Hi-C data.

Inspired by NMF, our EnHiC model uses similar notions of rank-1 features and matrix factorization. However, our model is different from NMF in the following aspects. First, our model attempts to decompose a set of submatrices, instead of a full matrix. In the decomposition step, it searches for a rank-1 solution that fits all submatrices. Here we limit the rank to 1 to bypass the problem of picking the appropriate number of ranks in a low-rank solution. Second, our model optimizes the rank-1 matrix decomposition via the *Decomposition & Reconstruction Block* in the GAN framework. The difference between the input Hi-C matrix and its rank-1 approximation is characterized by a loss function consisting of the L2 MSE loss and structural dissimilarity.

We demonstrated the performance of our EnHiC model using Hi-C datasets on three human cell lines. Overall, our EnHiC model evidently outperformed two other GAN-based methods, Deephic and HiCSR, achieving low prediction errors and high reproducibility scores when compared with the true high-resolution data. Moreover, EnHiC model is capable of recovering high-resolution Hi-C matrices across different cell types and from insufficiently sequenced input data. Additionally, we demonstrated that EnHiC-predicted matrices facilitated more accurate and precise detection of TADs and fine-scale chromatin interactions.

We envision a few possible extensions and future directions based on this work. First, EnHiC uses SCN normalization in the pre-processing step. The SCN normalization helps to reduce systematic biases in Hi-C data, and rescales the intensity values to real numbers between [0,1]. It is possible to add alternative options of other Hi-C normalization methods in the implementation. And we do not expect the choice of normalization methods to have a major impact on the model performance. Second, EnHiC requires the input matrices to be symmetric. In our experiments, when dividing the entire Hi-C matrix into small submatrices, we merged two on-diagonal submatrices with one off-diagonal matrix to generate a symmetric matrix. This divide-and-merge strategy may cause artifacts at the edges of the submatrices. One possible future extension is to build a paired layer that simultaneously estimates the row and column vectors to relax the symmetry requirement. Third, to effectively extract multi-scale rank-1 features, large input matrices are recommended. In the current setting, we used 400 × 400 submatrices to achieve the desired enhancement factor of 16. Increasing the dimension of the input matrices would require more memory allocation and result in a heavier computation load. One possible future extension is to build a distributed implementation to mitigate the burden on each node.

## Supplementary Material

btab272_Supplementary_Data
