# Supplementary material for: EnHiC: learning fine-resolution Hi-C contact maps using a generative adversarial framework
Source: Bioinformatics. 2021 Jul 12;37(Suppl 1):i272–9. doi: 10.1093/bioinformatics/btab272 (PMC8382278; doi:10.1093/bioinformatics/btab272)
Supplement: btab272_Supplementary_Data [file btab272_supplementary_data.pdf]

Supplementary Information for "EnHiC: Learning fine-resolution Hi-C contact maps using a generative adversarial framework"

Yangyang Hu and Wenxiu Ma

1 Supplementary method

1.1 Details of the convolutional blocks used in the EnHiC model

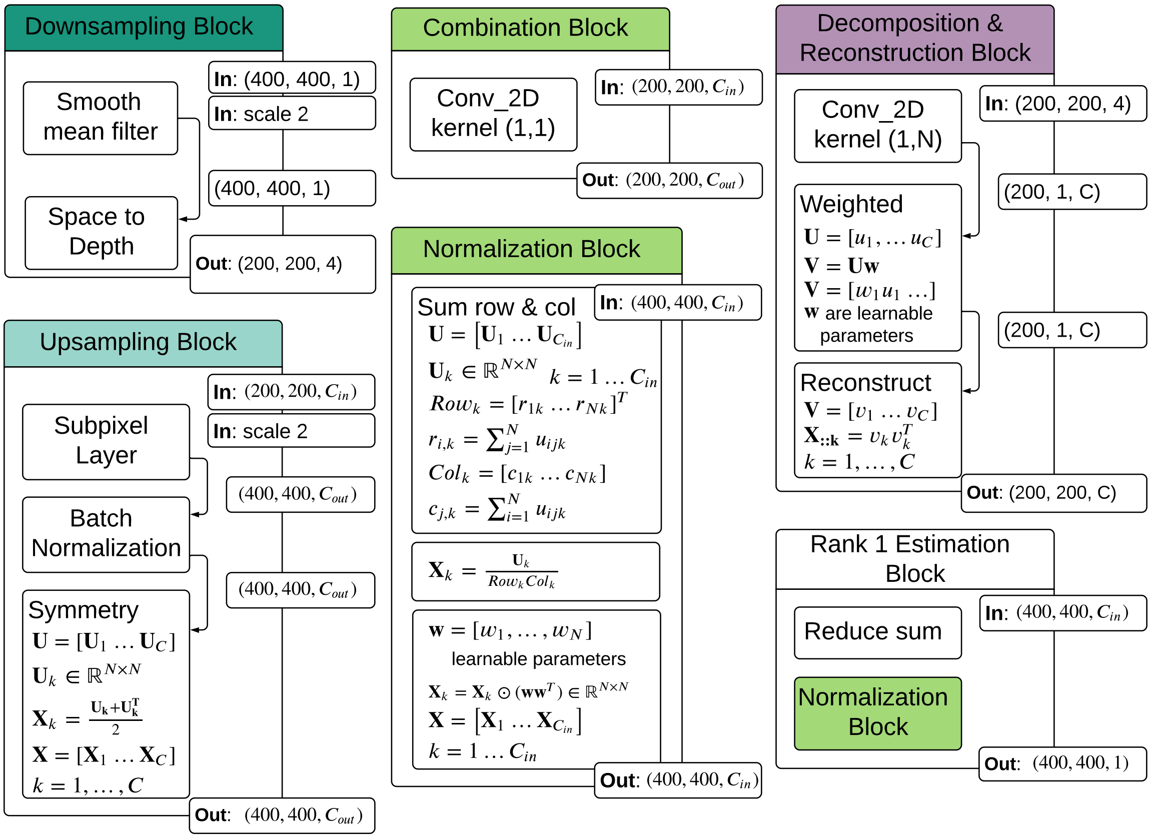

Supplementary Figure S1: Details of the convolutional blocks used in EnHiC

Downsampling Block

The downsampling block shrinks the height/width of the matrix and rearranges the blocks of spatial data into depth (channel).

Algorithm 1: Downsampling Block

Input: $\mathbf{U} \in \mathbb{R}^{N \times N \times c_{in}}$, ratio: r

Output: $\mathbf{X} \in \mathbb{R}^{\frac{N}{r} \times \frac{N}{r} \times c_{out}}$

1 Smooth the matrix by mean filtering: $\mathbf{X} = conv(\mathbf{U}), \mathbf{X} \in \mathbb{R}^{N \times N \times c_{in}}$, where the window size is $r \times r$

2 Rearrange blocks by *tf.nn.space_to_depth*: $\mathbf{X} = space\_to\_depth(\mathbf{X}, r)$

Decomposition & Reconstruction Block

This block aims to extract rank-1 matrices features.

Algorithm 2: Decomposition & Reconstruction Block

Input: $\mathbf{U} \in \mathbb{R}^{N \times N \times c_{in}}$

Output: $\mathbf{X} \in \mathbb{R}^{N \times N \times c_{out}}$

1 Decompose the matrices using a 2D convolution layer with kernel(1,N): $\mathbf{U} = conv2d(\mathbf{U}), \mathbf{U} \in \mathbb{R}^{N \times 1 \times c_{out}}$

2 Adjust weights of slices along the channels:

$$\mathbf{U} = [u_1, \dots u_{c_{out}}]$$

$$\mathbf{V} = \mathbf{U}\mathbf{w}, \mathbf{V} = [w_1 u_1 \dots]$$

where $\mathbf{w}$ are learnable parameters

3 Reconstruct the vectors into rank-1 matrix features:

$$\mathbf{V} = [v_1 \dots v_{c_{out}}]$$

$$\mathbf{X} = [\mathbf{X}_1 \dots \mathbf{X}_{c_{out}}], \mathbf{X}_\mathbf{k} = v_k v_k^T, k = 1, \dots, c_{out}$$

Combination Block

The combination block is a linear combination of the slices along the channels.

Algorithm 3: Combination Block

Input: $\mathbf{U} \in \mathbb{R}^{N \times N \times c_{in}}$

Output: $\mathbf{X} \in \mathbb{R}^{N \times N \times c_{out}}$

1 2D convolution layer with kernel(1,1): $\mathbf{X} = conv2d(\mathbf{U})$

Upsampling Block

This block employs a subpixel convolutional neural network layer to enhance the resolution from rank-1 matrix features.

Algorithm 4: Upsampling Block

Input: $\mathbf{U} \in \mathbb{R}^{N \times N \times c_{in}}$, ratio: r

Output: $\mathbf{X} \in \mathbb{R}^{(Nr) \times (Nr) \times c_{out}}$

1 Subpixel layer: $\mathbf{U} = subpixel(\mathbf{U}), \mathbf{U} \in \mathbb{R}^{(Nr) \times (Nr) \times c_{out}}$

2 Batch Normalization: $\mathbf{U} = BN(\mathbf{U})$

3 Symmetry: For each channel, average the slice with its transpose

$$\mathbf{U} = [\mathbf{U}_1 \dots \mathbf{U}_{c_{out}}], \mathbf{U}_k \in \mathbb{R}^{(Nr) \times (Nr)}, k = 1, \dots, c_{out}$$

$$\mathbf{X} = [\mathbf{X}_1 \dots \mathbf{X}_{c_{out}}], \mathbf{X}_k = \frac{\mathbf{U}_\mathbf{k} + \mathbf{U}_\mathbf{k}^\mathbf{T}}{2}, k = 1, \dots, c_{out}$$

Normalization Block

Similar to ICE (Imakaev et al., 2012), the normalization block learns the biases for rows and columns.

Algorithm 5: Normalization Block

Input: $\mathbf{U} \in \mathbb{R}^{N \times N \times c_{in}}$

Output: $\mathbf{X} \in \mathbb{R}^{N \times N \times c_{in}}$

1 Marginalize the slice by row and column:

$$\mathbf{U} = [\mathbf{U}_1 \dots \mathbf{U}_{C_{in}}], \mathbf{U}_k \in \mathbb{R}^{N \times N}, k = 1, \dots, c_{in}$$

$$Row_k = [r_{1k} \dots r_{Nk}]^T, r_{i,k} = \sum_{j=1}^{N} u_{ijk}$$

$$Col_k = [c_{1k} \dots c_{Nk}], c_{j,k} = \sum_{i=1}^{N} u_{ijk}$$

2 Normalize slice:

$$\mathbf{X}_k = \frac{\mathbf{U}_k}{Row_k Col_k}$$

3 Calculate the biases:

$$\mathbf{w} = [w_1, \dots, w_N]$$

$$\mathbf{X}_k = \mathbf{X}_k \odot (\mathbf{w}\mathbf{w}^T) \in \mathbb{R}^{N \times N}$$

$$\mathbf{X} = [\mathbf{X}_1 \dots \mathbf{X}_{C_{in}}], k = 1 \dots c_{in}$$

Where $\odot$ is element-wise multiplication and $\mathbf{w}$ are learnable parameters.

Rank-1 Estimation Block

This block sums all channels together and normalizes the output.

Algorithm 6: Rank-1 Estimation Block

Input: $\mathbf{U} \in \mathbb{R}^{N \times N \times c_{in}}$

Output: $\mathbf{X} \in \mathbb{R}^{N \times N \times 1}$

1 Sum all channels together to reduce the number of channels: $\mathbf{X} = sum(\mathbf{U}), \mathbf{X} \in \mathbb{R}^{N \times N \times 1}$

2 Pass the matrix to the Normalization Block

1.2 Generator

The architecture of the generator is illustrated below in Supplementary Figure S2:

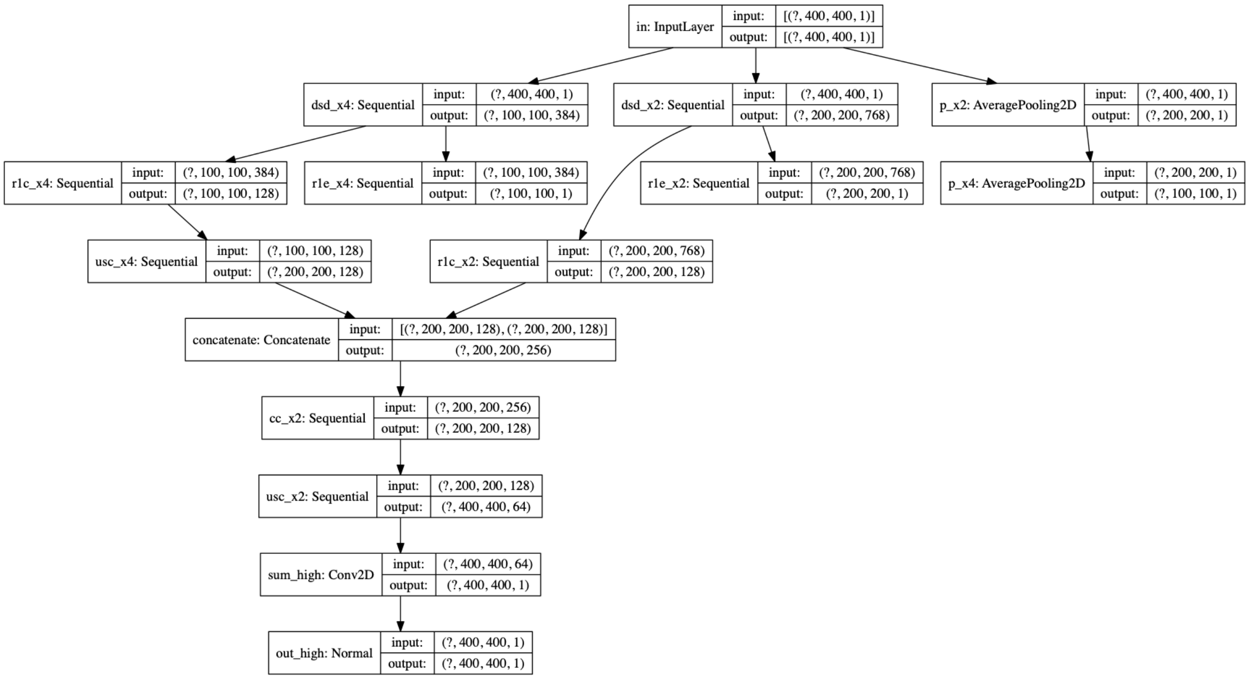

Supplementary Figure S2: The architecture of the generator model

1.3 Discriminator

The architecture of the discriminator is illustrated below in Supplementary Figure S3.:

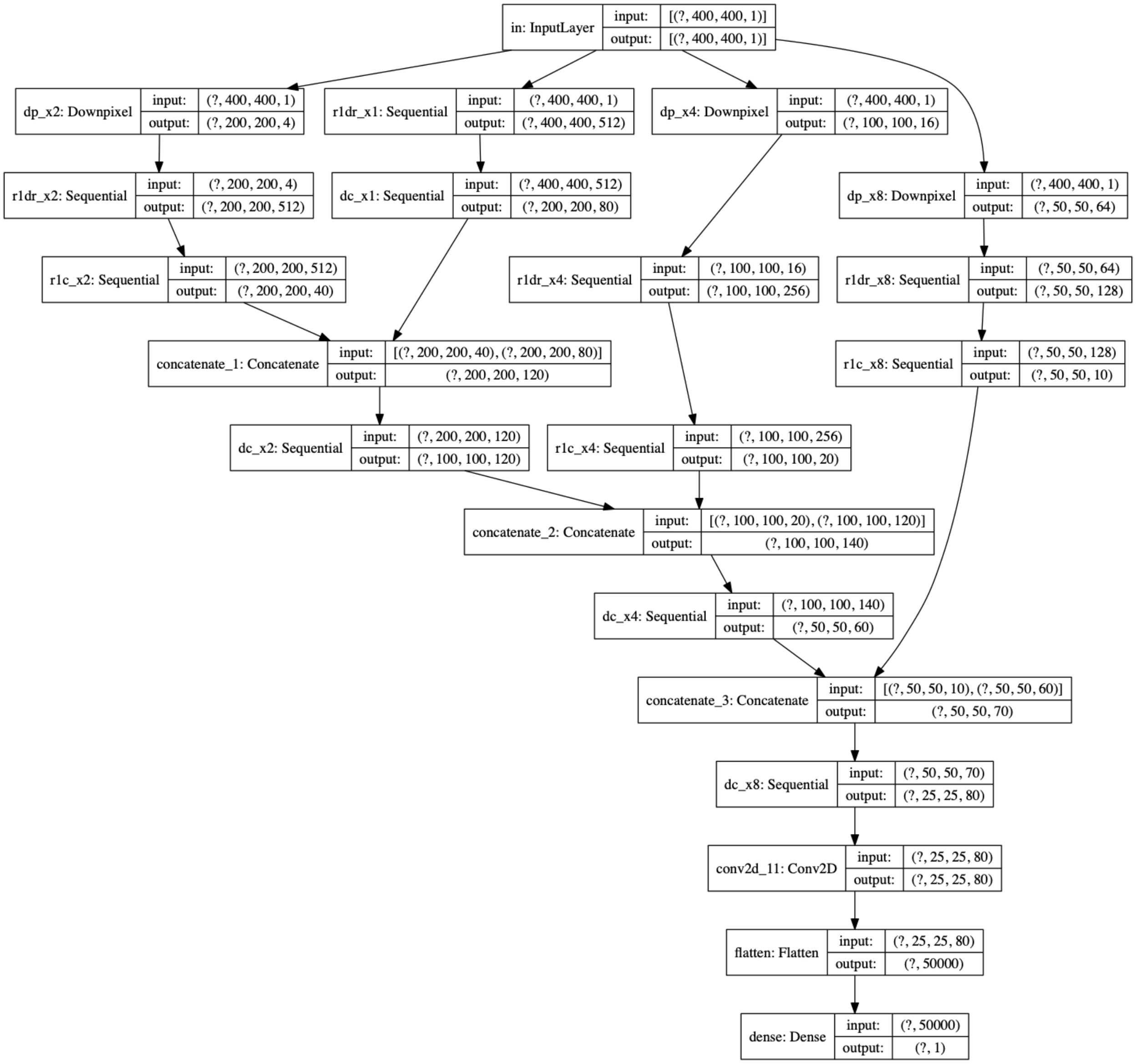

Supplementary Figure S3: The architecture of the discriminator model

1.4 Training and prediction

Supplementary Table S1 summarized the real Hi-C datasets used in the study. The filtered datasets were downloaded from the cooler database.

Cell line	RE	Assembly	Cis counts	Total counts
Rao2014 GM12878	MboI	hg19	2085711027	2884995088
Rao2014 IMR90	MboI	hg19	622282054	757393583
Rao2014 K562	MboI	hg19	473702480	617223144

Supplementary Table S1: Summary of the Hi-C datasets used in the study

Both training and prediction processes for the three models (EnHiC, Deephic, and HiCSR) were conducted using Intel Haswell CPU and NVIDIA Tesla K80 GPU wtih 128 GB of memory. The EnHiC model is implemented in Python with Tensorflow2.

We used chromosomes 1-16 for training, chromosomes 17 and 18 for hyper-parameters tuning, and chromosomes 19-22, and X for evaluation. The number of epochs for training is 300 and the parameters $\alpha_0 = 10, \alpha_1 = 0.1$. The runtime is approximately 85 hours (17 mins/epoch).

Below we plotted the MSE, DISSIM, and adversarial losses for the generator and discriminator in the training process in training dataset and validation dataset.

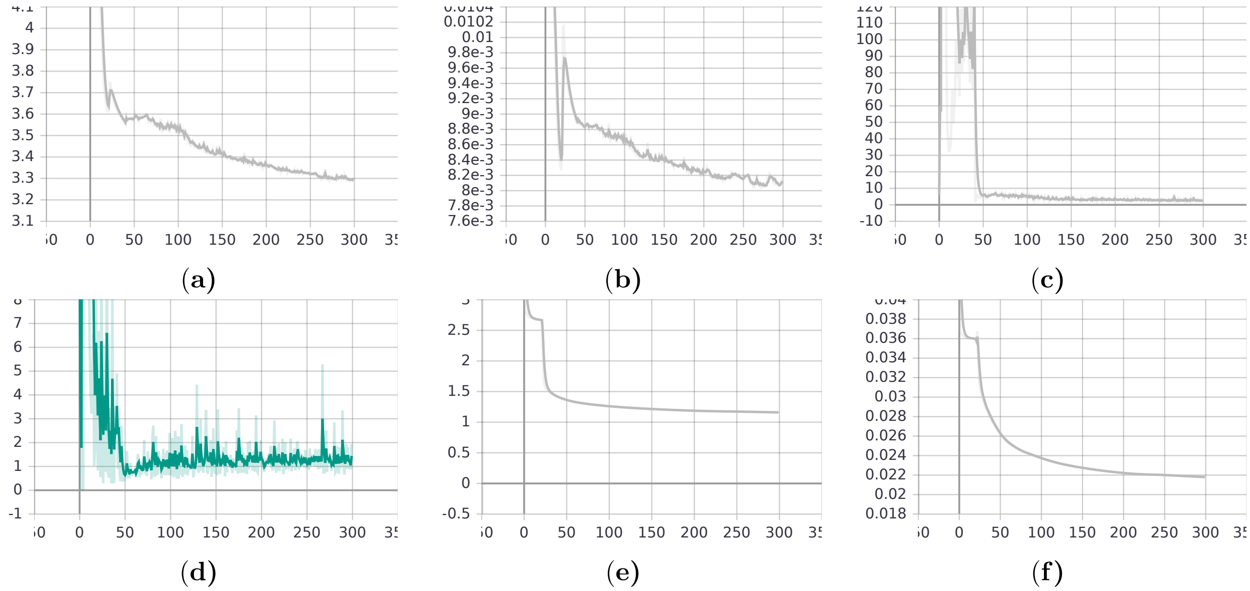

Supplementary Figure S4: **(a)** The MSE values of predictions at 10kb resolution in the training dataset, **(b)** The DISSIM values of predictions at 10kb resolution in the training dataset, **(c)** The adversarial loss values of generator in the training dataset, **(d)** The adversarial loss values of discriminator in the training dataset, **(e)** The weighted sum of MSE values of predictions at 20kb and 40kb resolutions in the training dataset. $MSE = \frac{MSE_{40kb}*4.0+MSE_{20kb}*16.0}{20.0}$, **(f)** The weighted of sum DISSIM values of predictions at 20kb and 40kb resolutions in the training dataset. $DISSIM = \frac{DISSIM_{40kb}*4.0+DISSIM_{20kb}*16.0}{20.0}$

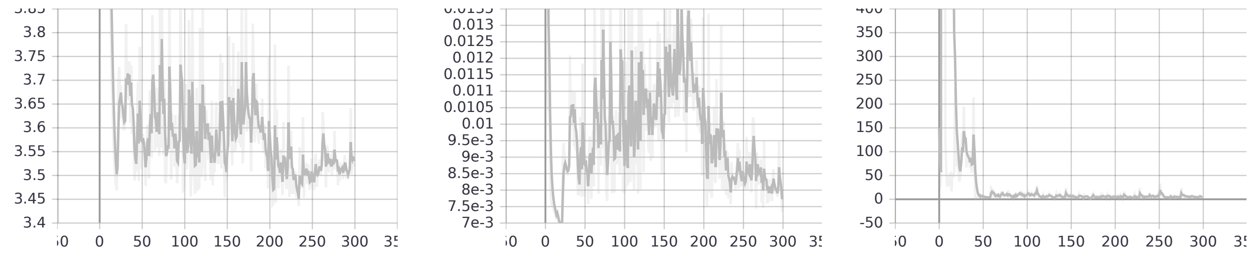

(**a**) (**b**) (**c**)

Supplementary Figure S5: **(a)** The MSE values of predictions at 10kb resolution in the validation dataset, **(b)** The DISSIM values of predictions at 10kb resolution in the validation dataset, **(c)** The adversarial loss values of predictions at 10kb resolution in the validation dataset.

	MAE			MSE		
Chr	Deephic	HiCSR	EnHiC	Deephic	HiCSR	EnHiC
19	3.46	4.46	0.29	665.6	2580.4	4.1
20	3.35	4.24	0.24	650.7	2453.1	2.5
21	5.71	8.57	0.52	966.6	5265.2	5.9
22	6.46	4.84	0.43	1417.1	1717.3	5.2
X	1.05	1.10	0.12	143.6	346.0	1.3

Supplementary Table S2: Evaluation of the high-resolution Hi-C matrices predicted by Deephic, HiCSR, and EnHiC. Each prediction result is compared against the ground truth; the MAE and MSE errors are reported.

2 Performance on different cell types

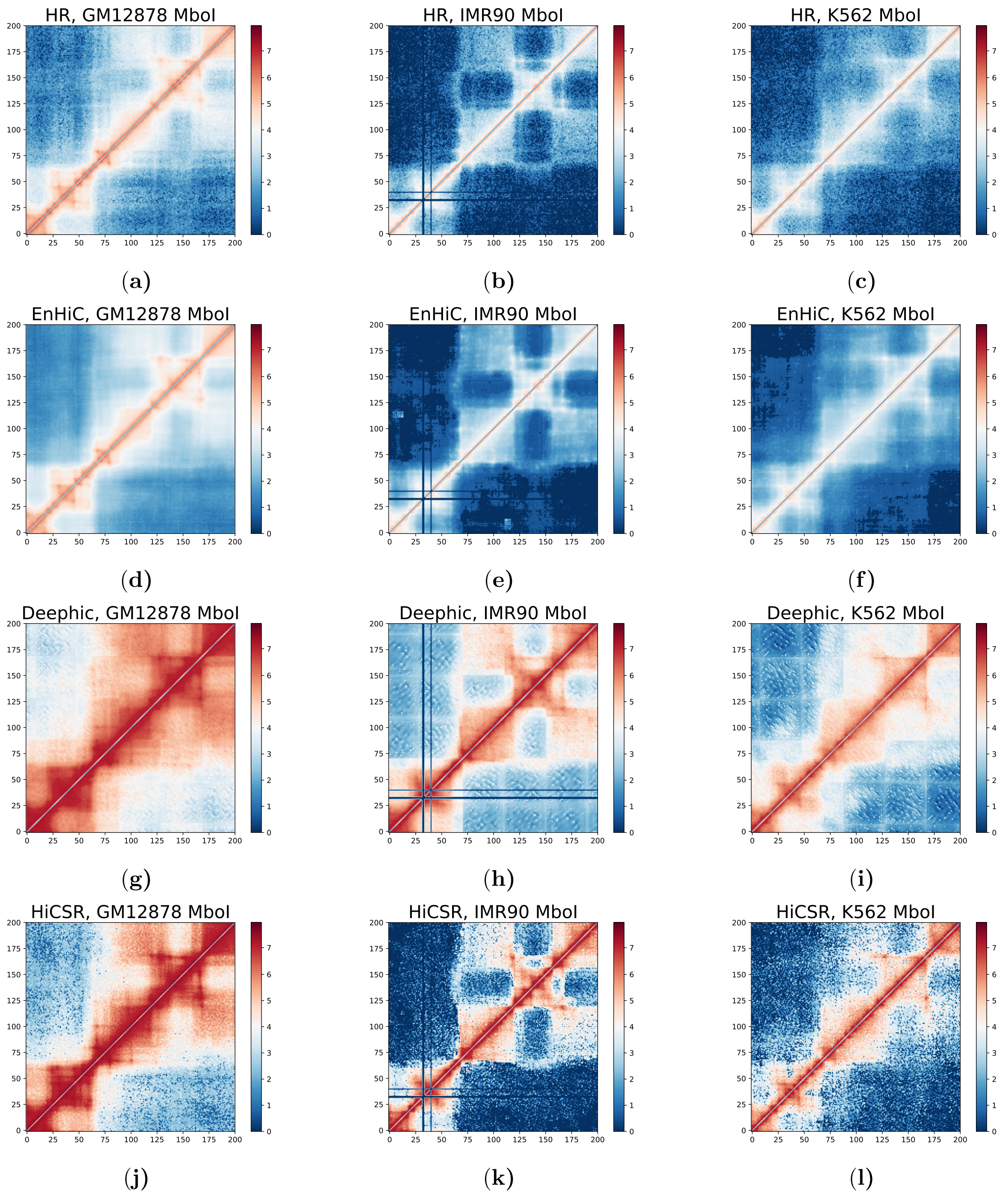

Supplementary Figure S6: Demo of model predictions on Hi-C data in three cell types (three columns: GM12878, IMR90, and K562) in the region of Chromosome 19 from 14Mbp to 16Mbp. The first row are the high-resolution Hi-C matrices (i.e., the ground-truth), the 2nd to 4th rows are the predictions from EnHiC, Deephic and HiCSR.

3 Analysis of different downsampling ratios

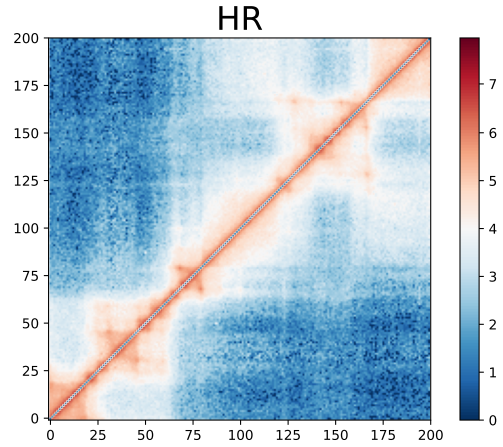

Supplementary Figure S7: The high-resolution Hi-C matrix at Chromosome 19 from 14Mbp to 16Mbp in human GM12878 cells. This is served as the ground truth for the model predictions in Supplementary Figures S8 and S9.

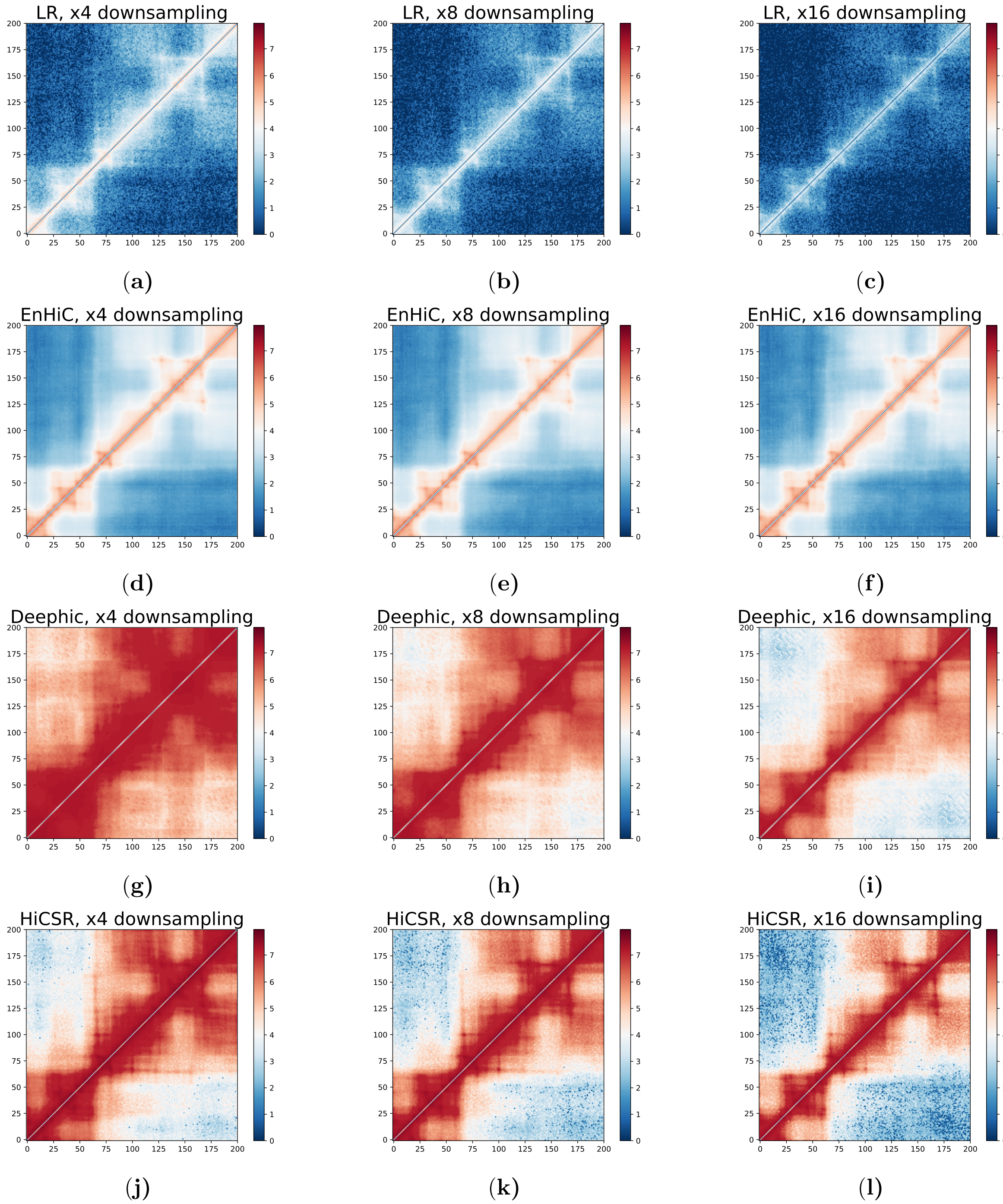

Supplementary Figure S8: Demo of model predictions on human GM12878 Hi-C data with three down-sampling ratios (4, 8, and 16) at Chromosome 19 from 14Mbp to 16Mbp. The first row are the downsampled Hi-C matrices, the 2nd to 4th rows are the model predictions from EnHiC, Deephic and HiCSR.

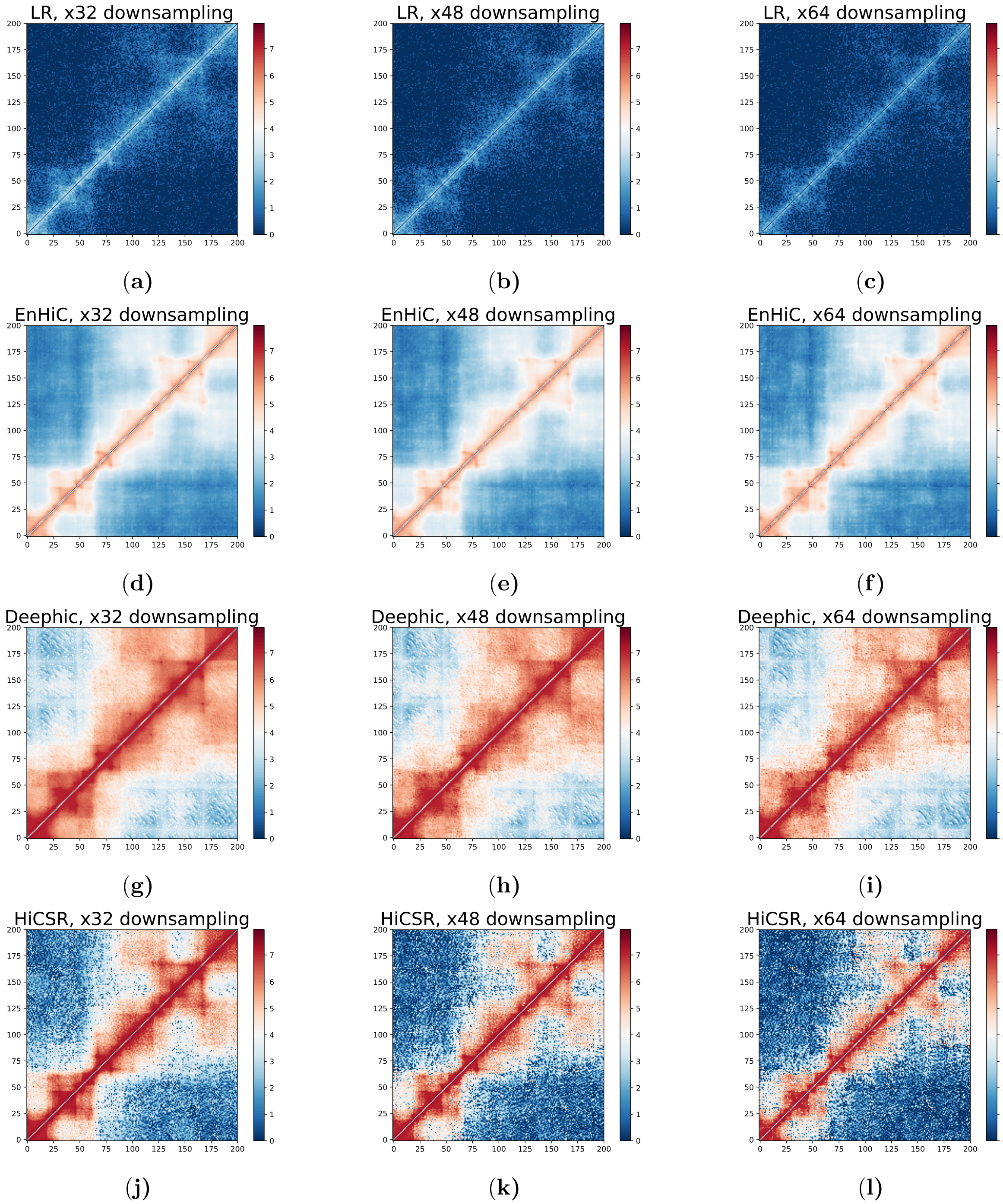

Supplementary Figure S9: Demo of model predictions on human GM12878 Hi-C data with three down-sampling ratios (32, 48, and 64) at Chromosome 19 from 14Mbp to 16Mbp. The first row are the downsampled Hi-C matrices, the 2nd to 4th rows are the model predictions from EnHiC, Deephic and HiCSR.

4 TAD detection

	Model	Jaccard score	# Intersection	# Model	# High
chr22	Deephic	60.00%	111	33	41
	HiCSR	55.45%	117	59	35
	EnHiC	**69.78**%	127	30	25
	Low Resolution	53.51%	99	33	53
chr21	Deephic	38.61%	61	63	34
	HiCSR	32.00%	56	80	39
	EnHiC	41.21%	68	70	27
	Low Resolution	**42.11**%	48	19	47
chr20	Deephic	61.59%	186	64	52
	HiCSR	58.02%	188	86	50
	EnHiC	**63.93**%	195	67	43
	Low Resolution	38.80%	116	61	122
chr19	Deephic	58.28%	190	61	75
	HiCSR	56.03%	195	83	70
	EnHiC	**65.71**%	207	50	58
	Low Resolution	50.63%	162	55	103
chr18	Deephic	42.44%	160	132	85
	HiCSR	42.52%	162	136	83
	EnHiC	**43.19**%	168	144	77
	Low Resolution	23.97%	76	72	169
chr17	Deephic	61.96%	259	78	81
	HiCSR	58.52%	261	106	79
	EnHiC	**68.70**%	281	69	59
	Low Resolution	50.12%	207	73	133
chrX	Deephic	**30.46**%	230	356	169
	HiCSR	26.46%	199	353	200
	EnHiC	28.97%	228	388	171
	Low Resolution	13.28%	66	98	333

Supplementary Table S3: The Jaccard Scores between TADs detected from the real high-resolution Hi-C data (10kb) and model predictions (EnHiC, Deephic, HiCSR) or low-resolution Hi-C input in chromosomes 17-22, and X. The last three columns are the intersection and differences. The # *Intersection* is the number of TADs detected in both high-resolution and predictions/low-resolution input. The # *High* is the number of TADs detected only in high-resolution Hi-C. The # *Model* is the number of TADs detected only in predictions/low-resolution input.

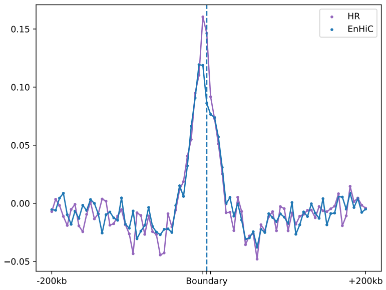

(**a**) CTCF

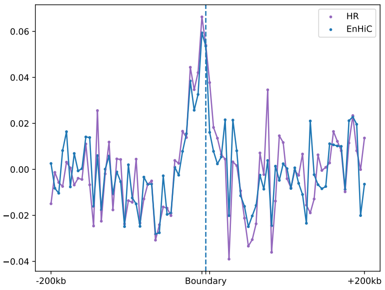

(**b**) Rad21

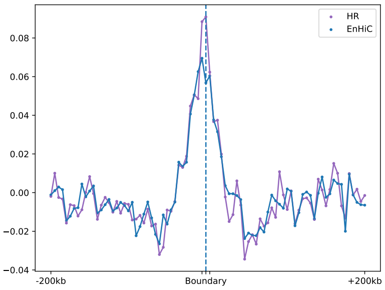

(**c**) Smc3

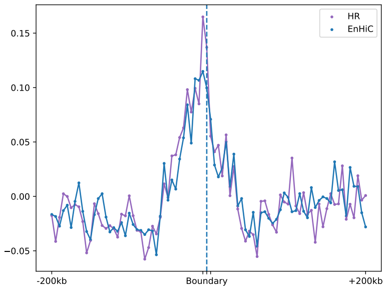

(**d**) PolII

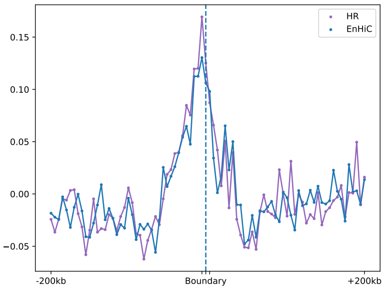

(**e**) PolII S2p

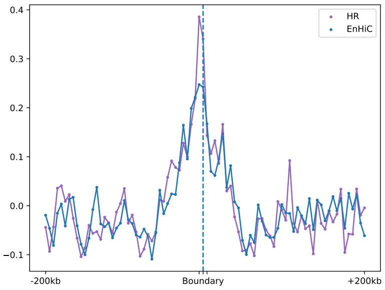

(**f**) PolII S5p

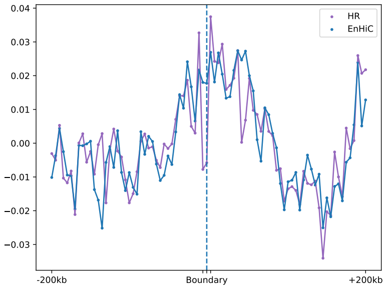

(**g**) H3K27me3

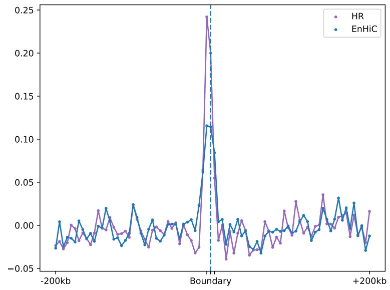

(**h**) H3K4me3

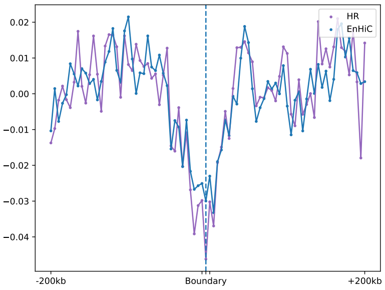

(**i**) H3K9me3

Supplementary Figure S10: ChIP-seq enrichment/depletion at TAD boundaries. ChIP-seq data were obtained from the ENCODE website, as documented in Supplementary Table S4.

Target	ChIP-seq file
CTCF	ENCFF271YKQ
Smc3	ENCFF235BXX
Rad21	ENCFF000WCT
H3K4me3	ENCFF818GNV
H3K9me3	ENCFF776OVW
H3K27me3	ENCFF594HSG
POlII	ENCFF368HBX
PolII S2p	ENCFF031RUV
PolII S5p	ENCFF002UPS

Supplementary Table S4: ChIP-seq datasets obtained from the ENCODE website.

5 Significant chromatin interactions

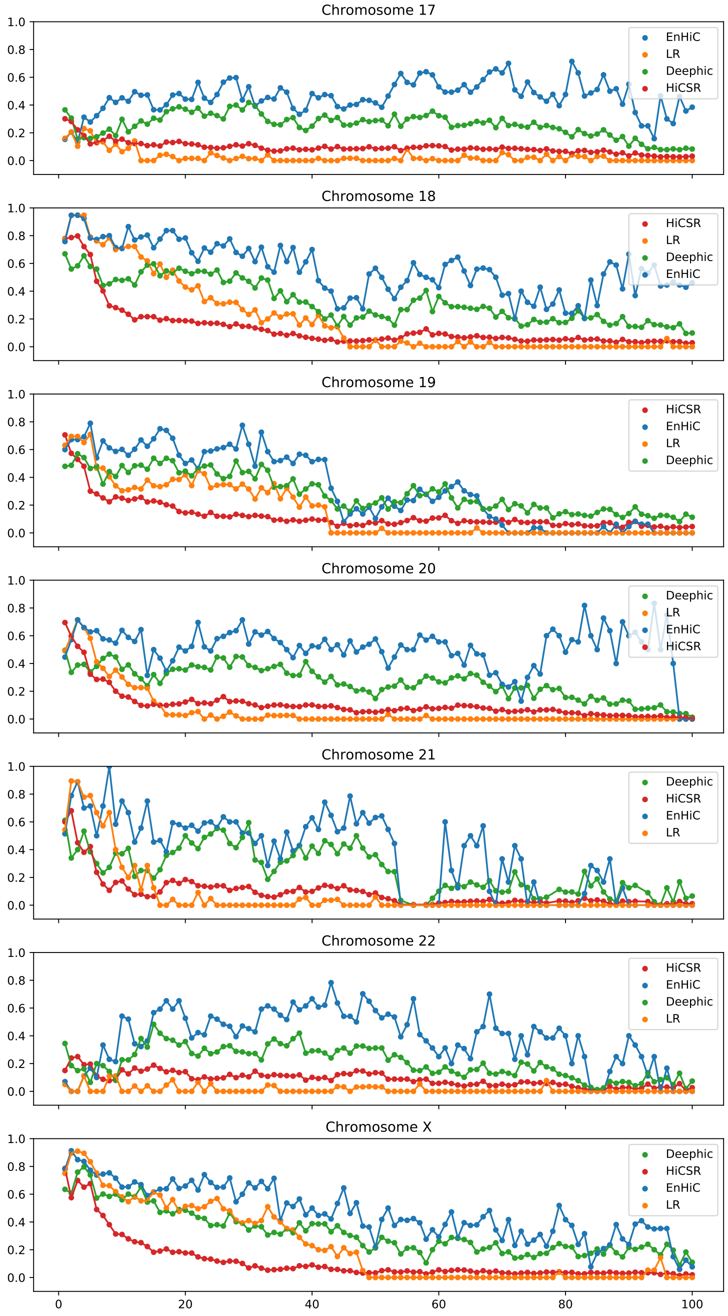

Supplementary Figure S11: The Jaccard Scores of significant interactions between high-resolution Hi-C and predictions/low-resolution input in seven chromosomes (17-22 and X). The LR represents the low-resolution Hi-C (40kb) downsampled from high-resolution Hi-C data. The x-axis (from 0 to 100) represents the genomic distance from 0 to 1000 kb.
